# Scientific output and organizational characteristics in Brazilian intensive care units: a multicenter cross-sectional study

**DOI:** 10.62675/2965-2774.20240006-en

**Published:** 2024-11-14

**Authors:** Thiago Tavares dos Santos, Luciano César Pontes de Azevedo, Antonio Paulo Nassar, Jorge Ibrain Figueira Salluh

**Affiliations:** 1 Instituto de Ensino e Pesquisa Hospital Sírio-Libanês São Paulo SP Brazil Instituto de Ensino e Pesquisa, Hospital Sírio-Libanês - São Paulo (SP), Brazil.; 2 Hospital Israelita Albert Einstein São Paulo SP Brazil Hospital Israelita Albert Einstein - São Paulo (SP), Brazil.; 3 A. C. Camargo Cancer Center São Paulo SP Brazil A. C. Camargo Cancer Center - São Paulo (SP), Brazil.; 4 Instituto D’Or de Pesquisa e Ensino Rio de Janeiro RJ Brazil Instituto D’Or de Pesquisa e Ensino - Rio de Janeiro (RJ), Brazil.

**Keywords:** Intensive care, Intensive care units, Research, Quality of health care, Benchmarking, Brazil

## Abstract

**Objective:**

To examine the associations between the scientific output of Brazilian intensive care units and their organizational characteristics.

**Methods:**

This study is a re-analysis of a previous retrospective cohort that evaluated organizational intensive care unit characteristics and their associations with outcomes. We analyzed data from 93 intensive care units across Brazil. Intensive care units were assessed for scientific productivity and the effects of their research activities, using indicators of care for comparison. We defined the most scientifically productive intensive care units as those with numerous publications and a SCImago Journal Rank score or an H-index above the median values of the participating intensive care units.

**Results:**

Intensive care units with more publications, higher SCImago Journal Rank scores and higher H-index scores had a greater number of certified intensivists (median of 7; IQR 5 - 10 *versus* 4; IQR 2 - 8; with p < 0.01 for the comparison between intensive care units with more *versus* fewer publications). Intensive care units with higher SCImago Journal Rank scores and H-index scores also had a greater number of fully implemented protocols (median of 8; IQR 6 - 8 *versus* 5; IQR 3.75 - 7.25; p < 0.01 for the comparison between intensive care units with higher *versus* lower SCImago Journal Rank scores).

**Conclusions:**

Scientific engagement was associated with better staffing patterns and greater protocol implementation, suggesting that research activity may be an indicator of better intensive care unit organization and care delivery.

## INTRODUCTION

In recent decades, several clinical studies have generated robust evidence for care delivery in intensive care units (ICUs).^(1,2)^ However, the translation of evidence-based medicine into better outcomes depends on the optimal implementation of research findings.^(3-7)^ In addition to producing the evidence necessary for the development of science, there is current evidence from several medical specialties that conducting research can improve the care of patients not directly involved in studies, and possibly increase the quality of care provided by the institutions where these studies are performed.^(8-12)^

The association between research activity and the quality of care has been demonstrated in specialties such as surgery, cardiology, and oncology.^(13-15)^ A large multicenter cohort study involving patients with ovarian cancer demonstrated that treatment at nonresearch centers was associated with an increased risk of death compared with treatment at research institutions.^(13)^ A systematic review evaluating studies in several research areas concluded that institutions and professionals who participated in studies had better clinical outcomes, adherence to guidelines, and increased use of evidence-based practices.^(16)^

Over the past 30 years, outcomes have significantly improved for ICU patients following evidence-based care guidelines,^(17-20)^ particularly those suffering from acute respiratory failure, acute respiratory distress syndrome (ARDS),^(21-23)^ or sepsis.^(24)^ Adherence to protocols and clinical pathways for treating these conditions is associated with fewer ICU-acquired complications and lower mortality rates. However, despite increases in research and publications on critical illness, few studies have examined the associations between clinical research activity and ICU organization indicators, especially in low- and middle-income countries (LMICs).

Our hypothesis was that a higher scientific output is associated with better ICU organizational characteristics, namely, the process of care measures and staffing, in a large sample of Brazilian ICUs. Our goal was to examine the associations between the scientific output of Brazilian intensive care units and their organizational characteristics.

## METHODS

### Study design and population

This study is a secondary analysis of the ORganizational CHaractEeriSTics in cRitical cAre (ORCHESTRA)^(25)^ study. The ORCHESTRA study is a continuous enrollment multicenter cohort study (since 2013) that aims to evaluate the associations between critical care organization characteristics and outcomes in critically ill patients. The ORCHESTRA II study was performed in 93 Brazilian ICUs from 55 hospitals during 2014 and 2015, in which clinical data from patients and data on ICU characteristics were gathered according to a specific survey.^(25)^ Local Ethics Committees and the Brazilian National Ethics Committee (CAAE: 19687113.8.1001.5249) waived the need for patients’ informed consent. General and specialized ICUs were included in the study, with the exception of those exclusively dedicated to cardiovascular patients.

All patient data were deidentified and extracted from the Epimed Monitor ICU Database^®^ software, which is a cloud-based ICU management system for quality improvement, benchmarking, and case-mix evaluation.^(26)^ Routinely collected data included demographics, admission diagnosis, comorbidities based on the Charlson Comorbidity Index (CCI), performance status (PS) in the week before hospital admission, Simplified Acute Physiological Score 3 (SAPS 3), Sequential Organ Failure Assessment (SOFA) score at admission, use of organ support and ICU/hospital outcomes.^(26)^

The organizational characteristics of these ICUs were collected for the ORCHESTRA II^(25)^ study via a structured survey applied in 2015 through interviews (in person or by telephone) with medical directors and/or nurses from the participating centers. Detailed data on the ORCHESTRA II study are published elsewhere.^(25)^ The data surveyed included ICU staffing patterns and a set of ten prespecified clinical protocols aimed at preventing ICU-acquired complications and treating acute illnesses.^(25)^ We also assessed nonphysician staff members’ autonomy by surveying the chief nurse, as previously described.^(26)^

### Scientific output

**Figure 1:**
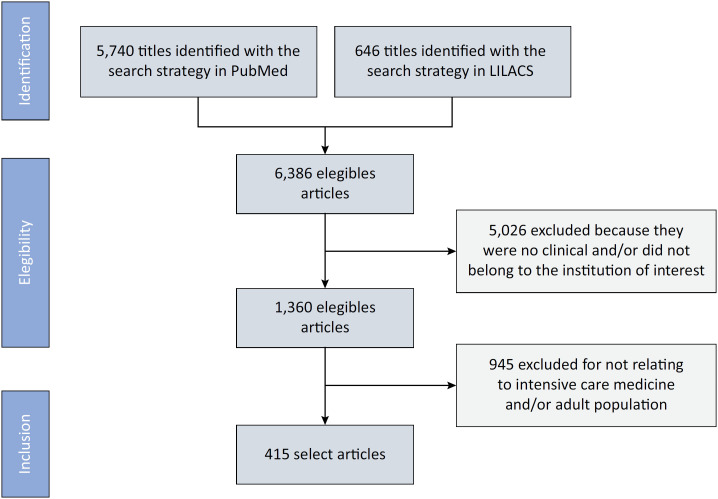
Study selection process.

We used the number of peer-reviewed publications as a proxy for scientific output. Data regarding scientific output were retrospectively obtained (in February 2020) through the construction of a search strategy for the PubMed and *Literatura Latino-americana e do Caribe em Ciências da Saúde* (LILACS) databases (Figure 1). The objective was to identify publications relative to studies carried out during the years 2014 and 2015, when the ORCHESTRA II study was conducted. Therefore, considering an arbitrary interval between conducting the research and its publication, we searched the literature for the period between 2014 and 2017. We identified and registered the total number of articles published in peer-reviewed scientific journals. Searches were performed by one author in the PubMed and LILACS databases and were verified by other authors when any uncertainty existed. Considering the unparalleled relevance and breadth of coverage offered by PubMed and the local representativeness of LILACS, coupled with our stringent requirement for peer-reviewed sources, we determined that extending our search to include other databases or gray literature would not significantly enhance the quality or relevance of our research.

Data regarding scientific activity were retrospectively obtained through the construction of a search strategy in peer-reviewed publication databases.

Furthermore, we utilized the advanced search feature on PubMed to search specifically for the name of the hospital associated with the study's ICU. This was part of our broader exploratory search strategy aimed at thoroughly understanding the topic without being confined to a rigid protocol. This flexible approach enabled us to discover a wide range of sources and types of information that a more structured, systematic method might not have captured. By focusing our search on the hospital of interest, we were able to identify the largest possible collection of relevant articles for our research (Appendix 1).

The literature search results were manually screened by the authors to identify the publications and confirm their sources as from the ICUs participating in the ORCHESTRA study.^(25)^ Confirmation included contact with the authors. In the case of institutions in which there was more than one ICU participating in the ORCHESTRA II^(25)^ study, the name of the ICU coordinator listed as a coauthor was used to determine the association with the publication. The total number of articles identified in the PubMed search for each ICU was added, creating a continuous variable. The same process was carried out in the LILACS database. The quality of these publications was assessed by the impact factor of the journals (H-index and the SCImago Journal Rank)^(27,28))^ in the year of the publication.

### Impact factors and qualification of scientific output

We evaluated the impact of the publications via the H-index and the SCImago Journal Rank of the journal where each article was published. The H-index is a measure of the scientific influence of academic journals that considers the number of citations of that journal and the importance of those citations. For the construction of the H-index continuous variable, the highest H-index among all publications was used.^(27)^

The SCImago Journal Rank score is a measure of the scientific influence of academic journals that considers the number of citations of that journal and the importance of those citations. The SCImago Journal Rank score is a free journal metric, an alternative to the impact factor (IF), and can be used for journal comparisons. The sum of the SCImago Journal Rank score of all publications was calculated for each ICU, thus generating a continuous variable.^(28)^

All ICUs were considered to have at least one publication, which was the index paper from the ORCHESTRA II study.^(25)^

We defined the most scientifically productive ICUs as those with several publications, a SCImago Journal Rank score or an H-index above the median values of the participating ICUs.

### Intensive care unit organization and outcomes

We evaluated proxies of quality of care via structure and process metrics. The measures were previously defined^(25)^ and included the following:

- Process measures: the number of fully implemented protocols.
- Staffing/structure: number of physicians certified by the *Associação de Medicina Intensiva Brasileira* (AMIB), the presence of a clinical pharmacist and the nursing autonomy score.

### Statistical analysis

Standard descriptive statistics and inferential statistical methods were applied. The qualitative variables (the presence of a clinical pharmacist) are presented as the distributions of absolute and relative frequencies and were compared with the chi-square test. Quantitative variables (the number of fully implemented protocols, number of certified physicians and nursing autonomy score) are presented as medians and interquartile ranges (IQR) and were compared with the Mann-Whitney test.

The alpha error was previously fixed at 5% for the null hypothesis, and statistical processing was performed using BioStat Programs version 5.3 and Statistical Package for the Social Sciences (SPSS) version 27.

## RESULTS

The characteristics of the ICUs included in the ORCHESTRA II study are summarized in table 1. After the initial search in PubMed and the triage of individual citations, a total of 415 articles met the inclusion criteria. Considering that the publication of the ORCHESTRA II article was scored for all participating ICUs, the mean number of publications per ICU was 5.45 articles (standard deviation: 12.79), whereas the median (IQR) were 2; IQR 2 - 4 articles, respectively. The minimum number of publications during the study period was one, and the maximum was 101. Fifty-six ICUs had two or fewer publications, and only 37 ICUs had more than two publications.

**Table 1:** Characteristics of the hospitals participating in the OSCHESTRA II study

Hospital type		
	Private	31 (56)
	State	10 (18)
	Philanthropic	14 (26)
Hospital beds		234 ± 134,202 (60 - 1,020)
	< 150	21 (38)
	150 - 300	24 (44)
	> 300	10 (18)
Number of ICUs in each hospital		
	1	20 (36)
	2 or 3	21 (38)
	> 3	14 (26)
ICU beds/hospital beds*		15.15 ± 6.7
Step-down unit		
	Yes	21 (38)
	No	34 (62)
Emergency department		
	Yes	48 (87)
	No	3 (5)
	Referral unit	4 (7)
Health care quality certification†		
	None	19 (34)
	National	20 (36)
	International	16 (30)
Rapid Response Teams		
	Yes	32 (58)
	No	23 (42)
Critical Care Fellowship		
	Yes	36 (66)
	No	19 (34)

ICU - intensive care unit.

* ICU beds/hospital beds * 100;

† Brazilian National Accreditation Organization (ONA), Joint Commission International (JCI) and Accreditation Canada Internation (ACI). Continuous variables are reported as the means ± standard deviations and medians (ranges).

The searches performed in the LILACS database resulted in a total of 646 articles; however, only one article met the inclusion criteria of this study. This article was already present in the search carried out in the PubMed database.

Regarding the impact, the H-index variable presented a mean value of 311.9, a median of 197, and IQR values of 197 and 987, respectively. The SCImago Journal Rank score variable presented a mean value of 14,379, with a median of 7,233 and IQR of 3.94 and 157.4, respectively.

Tables 2, 3 and 4 describe the organization variables of the intensive care units participating in the study and their associations with the scientific output.

**Table 2:** Intensive care unit characteristics according to the total number of published articles

	Peer-reviewed publications ≤ 2 (n = 56)	Peer-reviewed publications > 2 (n = 37)	p value
Number of fully implemented care protocols	5 (4 - 8)	7 (5 - 8)	0.18
Nursing autonomy score	6 (4 - 9)	7 (3 - 9)	0.73
Physicians certified by the *Associação de Medicina Intensiva Brasileira*	4 (2 - 8)	7 (5 - 10)	< 0.01
Presence of clinical pharmacist	32 (57.1)	21 (56.7)	0.97

The variable peer-reviewed publication is dichotomized by the median (Group 1 = value less than or equal to the median; Group 2 = value greater than the median). Numerical variables are presented as medians (interquartile ranges), and the presence of a pharmacist is presented as a number (%).

**Table 3:** Intensive care unit characteristics according to the SCImago Journal Rank score

	SJR ≤ 7.233 (n = 52)	SJR > 7.233 (n = 41)	p value
Number of fully implemented care protocols	5 (3.75 - 7.25)	8 (6 - 8)	< 0.01
Nursing autonomy score	7 (3.75 - 9)	6 (4 - 8)	0.67
Physicians certified by the *Associação de Medicina Intensiva Brasileira*	4 (2 - 7)	8 (5 - 13)	< 0.01
Presence of clinical pharmacist	28 (53.8)	25 (61.0)	0.63

SJR - SCImago Journal Rank. The SCImago Journal Rank variable is dichotomized by the median (Group 1 = value less than or equal to the median; Group 2 = value greater than the median). Numerical variables are presented as medians (interquartile ranges), and the presence of a pharmacist is presented as a number (%).

**Table 4:** Intensive care unit characteristics according to the H-index of the journal in which the article was published

	H-index ≤ 197 (n = 56)	H-index > 197 (n = 37)	p value
Number of fully implemented care protocols	5 (4 - 7.25)	8 (5 - 8)	0.01
Nursing autonomy score	6 (3 - 9)	7 (4 - 9)	0.77
Physicians certified by the *Associação de Medicina Intensiva Brasileira*	4 (2 - 7)	8 (5 - 14)	< 0.01
Presence of clinical pharmacist	30 (53.6)	23 (62.2)	0.54

The H-index variable is dichotomized by the median (Group 1 = value less than or equal to the median; Group 2 = value greater than the median). Numerical variables are presented as medians (interquartile ranges), and the presence of a pharmacist is presented as a number (%).

### Characteristics of intensive care units analyzed through publication volume, the SCImago Journal Rank Score, and the Journal H-index

The group of ICUs with publications above the median of the total number of publications (2 papers) had more physicians certified by the AMIB (a median of 7, IQR: 5 - 10) than did the group of ICUs with only one publication (a median of 4, interquartile range: 2 - 8, p < 0.01). In addition, the group of ICUs with publications in journals with a SCImago index higher than 7.23 (the median of the study) had a greater number of implemented clinical protocols (those aimed at preventing ICU-acquired complications and treating acute illnesses), with a median of 8 protocols (IQR 6 - 8) *versus* 5, IQR 3.75 - 7.25, p < 0.01. These ICUs also had more physicians certified by the AMIB than did the group of ICUs with publications in journals with a SCImago index lower than the median (median = 8, IQR 5 - 13.4, IQR 2 - 7, p < 0.01).

The group of ICUs that had publications in journals with H-index scores higher than the median of 197 had a greater number of implemented care protocols (median = 8, IQR: 5 - 8 *versus* - 5, IQR 4 - 7.25, p < 0.01); and more physicians certified by the AMIB (median = 8, IQR: 5 - 14 *versus* 4, IQR 2 - 7, p < 0.01).

## DISCUSSION

In this large multicenter study of Brazilian ICUs, we demonstrated that ICUs with more scientific outputs had more clinical protocols implemented and better staffing patterns (namely, more intensivists) than did ICUs with lower scientific outputs.

In this study, we used surrogates of processes and organizations in line with those that are currently recommended^(29-31)^ and that have been extensively validated.^(25,26,32-35)^ Soares et al. demonstrated in a study involving 78 Brazilian ICUs that protocol implementation by a multidisciplinary team (a combination of process and coordination of care by ICU staff) was associated with better survival rates.^(17)^ In addition, there is evidence that better ICU staffing and protocol implementation are strongly associated with better adherence to protective ventilation^(36)^ and sedation goals in mechanical ventilation MV patients.^(37)^

Interestingly, our findings demonstrate that for ICUs involved in clinical research, the potential organizational and outcome benefits are systemic and not limited to patients participating in clinical studies. It is, of course, legitimate to argue that ICUs with better staffing are potentially those with more resources and therefore will be more prone to perform clinical research. We believe that better processes of care (protocol implementation) demonstrate that, beyond causal relationships, ICUs involved in research are engaged in a process of optimizing standards of care (staffing and evidence-based protocols). Such conditions are ultimately associated with better outcomes.

Although previously not described for intensive care units, our findings are in line with the evidence found in other specialties, such as cardiology, neurology, and oncology. In these scenarios, compliance with quality-of-care indicators was superior in care settings with greater scientific activity. The management of acute coronary syndrome, the treatment of ovarian neoplasia, and improvements in the diagnosis of clinical neurological syndromes are examples.^(13-15)^

Our study has several merits. This is a large multicenter study involving ICUs in an upper-middle income country where we captured organizational patterns that were previously validated as associated with quality of care and outcomes in ICUs in general^(25)^ and specific settings.^(37,38)^

Nevertheless, the present study has several limitations. First, there is a lack of consensus in the literature on methods for measuring scientific production and output, the retrospective nature of the methodological model used, and inherent selection bias, as the participating ICUs were those already collaborating at some level in multicenter studies. Thus, we did not have ICUs without any academic insertion or scientific output as a comparator. However, we can hypothesize that if such differences are present among those involved in research, they would be even larger if other centers were included. Additionally, we did not measure the impact of clinical research activities, such as the standardized mortality ratio and patient outcomes, on ICU performance measurements; thus, a direct association between clinical research and better prognosis could not be demonstrated.

## CONCLUSION

Brazilian intensive care units involved in clinical research demonstrated by increased number of publications, and publications with higher SCImago Journal Rank scores and H-index scores have better staffing patterns and clinical protocol implementation. These findings confirm the importance of investing in intensive care unit organization while creating appropriate conditions for advancing knowledge through clinical research.

## Appendix 1 Database search structure

**Table:**

Hospital	Search structure
*Hospital Agenor Paiva* - Salvador (BA), Brazil	"Hospital Agenor Paiva" OR "Agenor Paiva"
*Hospital Santa Helena* - Camaçari (BA), Brazil	"Hospital Santa Helena" OR ("Hospital Santa Helena") AND ("Camaçari") OR "Hospital Santa Helena, Camaçari"
*Hospital Anchieta Distrito Federal* - Taguatinga (DF), Brazil	"Hospital Anchieta Distrito Federal, Taguatinga" OR "Hospital Anchieta" OR "Hospital Anchieta Distrito Federal"
*Hospital Brasília* - Brasília (DF), Brazil	"Hospital Brasília"
*Hospital Santa Luzia Rede D’Or São Luiz DF* - Brasília (DF), Brazil	"Hospital Santa Luzia Rede D’Or São Luiz DF" OR "Hospital Santa Luzia Rede D’Or" OR "Hospital Santa Luzia" OR "Santa Luzia Rede D’Or"
*Hospital Unimed Vitória -* Vitória (ES), Brazil	"Hospital Unimed Vitoria" OR "Unimed Vitoria"
*Hospital Geral de Goiânia* - Goiânia (GO), Brazil	"Hospital Geral de Goiânia" OR "Hospital Geral Goiânia"
*Hospital de Câncer do Maranhão Tarquínio Lopes Filho* - São Luís (MA), Brazil	"Hospital de Câncer do Maranhão Tarquínio Lopes Filho" OR "Hospital Maranhão Tarquínio Lopes Filho"
*UDI Hospital* - São Luís(MA), Brazil	"Hospital UDI" OR "UDI hospital"
*Santa Casa de Caridade de Diamantina* - Diamantina (MG), Brazil	"Santa Casa Diamantina" OR "Caridade de Diamantina"
*Hospital das Clínicas, Universidade Federal de Minas Gerais* - Belo Horizonte (MG), Brazil	"Hospital" OR "Hospital das Clínicas" AND "universidade Federal de Minas Gerais"
*Hospital Universitário Lauro Wanderley* - João Pessoa (PB), Brazil	("Hospital Universitário Lauro Wanderley" OR "Hospital Lauro Wanderley" OR "Lauro Wanderley" OR ("Hospital Universitário da Universidade Federal da Paraiba")
*Hospital Esperança* - Recife (PE), Brazil	"Hospital Esperança"
*Hospital Esperança Olinda* - Olinda (PE), Brazil	"Hospital Esperança Olinda"
*Hospital São Marcos* - Recife (PE), Brazil	"Hospital São Marcos"
*Hospital Estadual Getúlio Vargas* - Rio de Janeiro (RJ), Brazil	"Hospital Estadual Getúlio Vargas" OR "Hospital Getúlio Vargas"
*Clínica São Vicente* - Rio de Janeiro (RJ), Brazil	"Clínica São Vicente"
*Hospital Estadual Adão Pereira Nunes* - Duque de Caxias (RJ), Brazil	"Hospital Estadual Adão Pereira Nunes"
*Hospital Estadual Carlos Chagas* - Rio de Janeiro (RJ), Brazil	"Hospital Estadual Carlos Chagas"
*Hospital Quinta D’Or* - Rio de Janeiro (RJ), Brazil	((("Hospital Quinta D’Or") OR ("Hospital Quinta Dor")) OR ("Quinta D’Or")) OR ("Quinta Dor")
*Hospital Barra D’Or* - Rio de Janeiro (RJ), Brazil	((("Hospital Barra D’Or") OR ("Hospital Barra Dor")) OR ("Barra D’Or")) OR ("Barra Dor")
*Hospital Copa D’Or* - Rio de Janeiro (RJ), Brazil	((("Hospital Copa D’Or") OR ("Hospital Copa Dor")) OR ("Copa D’Or")) OR ("Copa Dor")
*Hospital Caxias D’Or -* Duque de Caxias (RJ), Brazil	((("Hospital Caxias D’Or") OR ("Hospital Caxias Dor")) OR ("Caxias D’Or")) OR ("Caxias Dor")
*Hospital Norte D’Or* - Rio de Janeiro (RJ), Brazil	((("Hospital Norte D’Or") OR ("Hospital Norte Dor")) OR ("Norte Dor")) OR ("Norte D’Or")
*Hospital Oeste D’Or* - Rio de Janeiro (RJ), Brazil	"Hospital Oeste D’Or" OR "Hospital Oeste Dor" OR "Oeste D’Or" OR "Oeste Dor"
*Hospital Rios D'Or* - Rio de Janeiro (RJ), Brazil	((("Hospital Rios D’Or") OR ("Hospital Rios Dor")) OR ("Rios D’Or")) OR ("Rios Dor")
*Hospital Municipal Souza Aguiar*, Rio de Janeiro (RJ), Brazil	"Hospital Municipal Souza Aguiar" OR "Souza Aguiar" OR "Hospital Souza Aguiar" OR "HMSA"
*Hospital São Lucas* - Rio de Janeiro (RJ), Brazil	"Hospital São Lucas" OR "São Lucas"
*Hospital Unimed Costa do Sol* - Campos dos Goytacazes (RJ), Brazil	"Hospital Unimed Costa do Sol" OR "Hospital Costa do Sol" OR "Hospital Costa do Sol"
*Hospital Niterói D’Or* - Niterói (RJ), Brazil	"Hospital Niteroi D’Or" OR "Hospital Niteroi Dor" OR "Niteroi D’Or" OR "Niteroi Dor"
*Hospital Israelita Albert Sabin* - Rio de Janeiro (RJ), Brazil	"Hospital Israelita Albert Sabin" OR "Israelita Albert Sabin" OR "Hospital Albert Sabin"
*Samer Hospital* - Resende (RJ), Brazil	"Samer Hospital, Resende" OR "Samer"
*Hospital Estadual Alberto Torres* - São Gonçalo (RJ), Brazil	"Hospital Estadual Alberto Torres" OR "Hospital Alberto Torres" OR "Alberto Torres"
*Instituto Nacional de Câncer - HC II* - Rio de Janeiro (RJ), Brazil	"Instituto Nacional de Câncer" OR "INCA"
*Hospital Badim* - Rio de Janeiro (RJ), Brazil	"Hospital Badim" OR "Badim"
*Hospital Santa Rita, Santa Casa de Misericórdia de Porto Alegre* - Porto Alegre (RS), Brazil	("Santa Casa de Misericórdia de Porto Alegre") OR ("Hospital Santa Rita")
*Pavilhão Pereira Filho, Santa Casa de Misericórdia de Porto Alegre* - Porto Alegre (RS), Brazil	(("Santa Casa de Misericórdia de Porto Alegre") AND ("Pavilhão Pereira Filho")) OR (("Pavilhão Pereira Filho"))
*Hospital Dom Vicente Scherer, Santa Casa de Misericórdia de Porto Alegre* - Porto Alegre (RS), Brazil	(("Santa Casa de Misericórdia de Porto Alegre") AND ("Hospital Dom Vicente Scherer")) OR (("Hospital Dom Vicente Scherer"))
*Hospital Montenegro* - Montenegro (RS), Brazil	"Hospital Montenegro"
*Hospital São Francisco*, Ribeirão Preto (SP), Brazil	"Hospital São Francisco" AND "Ribeirão Preto"
*Hospital Vivalle* - São José dos Campos (SP), Brazil	"Hospital Vivalle"
*Fundação Pio XII - Hospital de Câncer de Barretos* – Barretos (SP), Brazil	"Hospital de Câncer de Barretos" OR "Fundação Pio XII"
*Hospital Alemão Oswaldo Cruz* - São Paulo (SP), Brazil	"Hospital Alemão Oswaldo Cruz" OR "Hospital Oswaldo Cruz"
*Hospital Israelita Albert Einstein* - São Paulo (SP), Brazil	("Hospital Israelita Albert Einstein") OR ("Hospital Albert Einstein")
*Hospital Sírio Libanês* - São Paulo (SP), Brazil	"Hospital Sírio Libanês" OR "Sírio Libanês"
*Hospital São Luiz - Unidade Assunção* - São Bernardo do Campo (SP), Brazil	"Hospital São Luiz" AND "Unidade Assunção"
*Hospital Sepaco* - São Paulo (SP), Brazil	"Hospital Sepaco"
*Hospital Santa Paula* - São Paulo (SP), Brazil	"Hospital Santa Paula"
*Hospital do Rim* - São Paulo (SP), Brazil	"Hospital do Rim" OR "Hrim"
*Rede D’Or São Luiz - Unidade Itaim* - São Paulo (SP), Brazil	(((("Rede Dor São Luiz" OR "Itaim")) OR ("Rede D’Or São Luiz" OR "Itaim")) OR ("São Luiz" OR "Itaim")) OR ("Rede D’Or" OR "Itaim")) OR ("Rede Dor" OR "Itaim")
*Hospital Samaritano* - São Paulo (SP), Brazil	"Hospital Samaritano"
*Hospital do Coração* – HCor - São Paulo (SP), Brazil	"Hospital do Coração" OR "HCor"
*Hospital Nove de Julho* - São Paulo (SP), Brazil	"Hospital Nove de Julho" OR "Hospital 9 de Julho"
*Hospital da Luz - Vila Mariana* - São Paulo (SP), Brazil	"Hospital da Luz"
*Rede D’Or São Luiz - Unidade Morumbi* - São Paulo (SP), Brazil	(((("Rede Dor São Luiz" AND "Morumbi")) OR ("Rede D’Or São Luiz" AND "Morumbi")) OR ("São Luiz" AND "Morumbi")) OR ("Rede D’Or"AND "Morumbi")) OR ("Rede Dor" AND "Morumbi")

In all searches, time (period 2014 to 2017) and language (English) filters were applied.

## References

[1] Deutschman CS, Neligan PJ (2019). Evidence-based practice of critical care.

[2] Kalassian KG, Dremsizov T, Angus DC (2002). Translating research evidence into clinical practice: new challenges for critical care. Crit Care.

[3] Pronovost P, Berenholtz SM, Needham DM (2008). Translating evidence into practice: a model for large scale knowledge translation. BMJ.

[4] Cabana MD, Rand CS, Powe NR, Wu AW, Wilson MH, Abboud PA (1999). Why don't physicians follow clinical practice guidelines? A framework for improvement. JAMA.

[5] Straus SE, Tetroe J, Graham I (2009). Defining knowledge translation. CMAJ.

[6] Dougherty D, Conway PH (2008). The "3T's" road map to transform US health care: the "how" of high-quality care. JAMA.

[7] Cooley ME, Biedrzycki B, Brant JM, Hammer MJ, Lally RM, Tucker S (2023). Translation of evidence-based interventions into oncology care settings: an integrative review. Cancer Nurs.

[8] Ozdemir BA, Karthikesalingam A, Sinha S, Poloniecki JD, Hinchliffe RJ, Thompson MM (2015). Research activity and the association with mortality. PLoS One.

[9] García-Romero A, Escribano A, Tribó JA (2017). The impact of health research on length of stay in Spanish public hospitals. Res Policy.

[10] Janni W, Kiechle M, Sommer H, Rack B, Gauger K, Heinrigs M, Steinfeld D, Augustin D, Simon W, Harbeck N, Friese K, ADEBAR Study Group (2006). Study participation improves treatment strategies and individual patient care in participating centers. Anticancer Res.

[11] Bennett WO, Bird JH, Burrows SA, Counter PR, Reddy VM (2012). Does academic output correlate with better mortality rates in NHS trusts in England?. Public Health.

[12] Majumdar SR, Roe MT, Peterson ED, Chen AY, Gibler WB, Armstrong PW (2008). Better outcomes for patients treated at hospitals that participate in clinical trials. Arch Intern Med.

[13] Pons J, Sais C, Illa C, Méndez R, Suñen E, Casas M (2010). Is there an association between the quality of hospitals’ research and their quality of care?. J Health Serv Res Policy.

[14] Downing A, Morris EJ, Corrigan N, Sebag-Montefiore D, Finan PJ, Thomas JD (2017). High hospital research participation and improved colorectal cancer survival outcomes: a population-based study. Gut.

[15] Are C, Caniglia A, Malik M, Smith L, Cummings C, Lecoq C (2018). Global variations in the level of cancer-related research activity and correlation to cancer-specific mortality: proposal for a global curriculum. Eur J Surg Oncol.

[16] Clarke M, Loudon K (2011). Effects on patients of their healthcare practitioner's or institution's participation in clinical trials: a systematic review. Trials.

[17] Soares M, Bozza FA, Angus DC, Japiassú AM, Viana WN, Costa R (2015). Organizational characteristics, outcomes, and resource use in 78 Brazilian intensive care units: the ORCHESTRA study. Intensive Care Med.

[18] Phillips C (2015). Relationships between duration of practice, educational level, and perception of barriers to implement evidence-based practice among critical care nurses. Int J Evid Based Healthc.

[19] Vranas KC, Scott JY, Badawi O, Harhay MO, Slatore CG, Sullivan DR (2020). The association of ICU acuity with adherence to ICU evidence-based processes of care. Chest.

[20] Levy MM, Rapoport J, Lemeshow S, Chalfin DB, Phillips G, Danis M (2008). Association between critical care physician management and patient mortality in the intensive care unit. Ann Intern Med.

[21] Neto AS, Barbas CS, Simonis FD, Artigas-Raventós A, Canet J, Determann RM, Anstey J, Hedenstierna G, Hemmes SN, Hermans G, Hiesmayr M, Hollmann MW, Jaber S, Martin-Loeches I, Mills GH, Pearse RM, Putensen C, Schmid W, Severgnini P, Smith R, Treschan TA, Tschernko EM, Melo MF, Wrigge H, de Abreu MG, Pelosi P, Schultz MJ, PRoVENT; PROVE Network investigator (2016). Epidemiological characteristics, practice of ventilation, and clinical outcome in patients at risk of acute respiratory distress syndrome in intensive care units from 16 countries (PRoVENT): an international, multicentre, prospective study. Lancet Respir Med.

[22] Parry SM, Berney S, Koopman R, Bryant A, El-Ansary D, Puthucheary Z (2012). Early rehabilitation in critical care (eRiCC): functional electrical stimulation with cycling protocol for a randomised controlled trial. BMJ Open.

[23] Laffey JG, Bellani G, Pham T, Fan E, Madotto F, Bajwa EK, Brochard L, Clarkson K, Esteban A, Gattinoni L, van Haren F, Heunks LM, Kurahashi K, Laake JH, Larsson A, McAuley DF, McNamee L, Nin N, Qiu H, Ranieri M, Rubenfeld GD, Thompson BT, Wrigge H, Slutsky AS, Pesenti A, LUNG SAFE Investigators and the ESICM Trials Group (2016). Potentially modifiable factors contributing to outcome from acute respiratory distress syndrome: the LUNG SAFE study. Intensive Care Med.

[24] Evans L, Rhodes A, Alhazzani W, Antonelli M, Coopersmith CM, French C (2021). Surviving Sepsis Campaign: International Guidelines for Management of Sepsis and Septic Shock 2021. Crit Care Med.

[25] Zampieri FG, Iwashyna TJ, Viglianti EM, Taniguchi LU, Viana WN, Costa R, Corrêa TD, Moreira CEN, Maia MO, Moralez GM, Lisboa T, Ferez MA, Freitas CE, de Carvalho CB, Mazza BF, Lima MF, Ramos GV, Silva AR, Bozza FA, Salluh JI, Soares M, ORCHESTRA Study Investigators (2018). Association of frailty with short-term outcomes, organ support and resource use in critically ill patients. Intensive Care Med.

[26] Zampieri FG, Salluh JI, Azevedo LC, Kahn JM, Damiani LP, Borges LP, Viana WN, Costa R, Corrêa TD, Araya DE, Maia MO, Ferez MA, Carvalho AG, Knibel MF, Melo UO, Santino MS, Lisboa T, Caser EB, Besen BA, Bozza FA, Angus DC, Soares M, ORCHESTRA Study Investigators (2019). ICU staffing feature phenotypes and their relationship with patients’ outcomes: an unsupervised machine learning analysis. Intensive Care Med.

[27] Alonso S, Cabrerizo FJ, Herrera-Viedma E, Herrera F (2009). h-Index: a review focused in its variants, computation and standardization for different scientific fields. J Informetr.

[28] Scimago Journal & Country Rank (SJR).

[29] Rhodes A, Moreno RP, Azoulay E, Capuzzo M, Chiche JD, Eddleston J, Endacott R, Ferdinande P, Flaatten H, Guidet B, Kuhlen R, León-Gil C, Martin Delgado MC, Metnitz PG, Soares M, Sprung CL, Timsit JF, Valentin A, Task Force on Safety and Quality of European Society of Intensive Care Medicine (ESICM) (2012). Prospectively defined indicators to improve the safety and quality of care for critically ill patients: a report from the Task Force on Safety and Quality of the European Society of Intensive Care Medicine (ESICM). Intensive Care Med.

[30] Pari V, Collaboration for Research Implementation, Training in Critical Care, Asia Africa ‘CCAA’ (2022). Development of a quality indicator set to measure and improve quality of ICU care in low- and middle-income countries. Intensive Care Med.

[31] Donabedian A (2005). Evaluating the quality of medical care. 1966. Milbank Q.

[32] Beane A, Salluh JI, Haniffa R (2021). What intensive care registries can teach us about outcomes. Curr Opin Crit Care.

[33] Lange DW, Dongelmans DA, Keizer NF (2017). Small steps beyond benchmarking. Rev Bras Ter Intensiva.

[34] Verburg IW, Holman R, Dongelmans D, de Jonge E, de Keizer NF (2018). Is patient length of stay associated with intensive care unit characteristics?. J Crit Care.

[35] Zampieri FG, Soares M, Salluh JI (2020). How to evaluate intensive care unit performance during the COVID-19 pandemic. Rev Bras Ter Intensiva.

[36] Midega TD, Bozza FA, Machado FR, Guimarães HP, Salluh JI, Nassar AP, Normílio-Silva K, Schultz MJ, Cavalcanti AB, Serpa A, CHECKLIST-ICU Investigators and the Brazilian Research in Intensive Care Network (BRICNet) (2020). Organizational factors associated with adherence to low tidal volume ventilation: a secondary analysis of the CHECKLIST-ICU database. Ann Intensive Care.

[37] Nassar AP, Zampieri FG, Salluh JI, Bozza FA, Machado FR, Guimarães HP (2019). Organizational factors associated with target sedation on the first 48 h of mechanical ventilation: an analysis of checklist-ICU database. Crit Care.

[38] Weled BJ, Adzhigirey LA, Hodgman TM, Brilli RJ, Spevetz A, Kline AM, Montgomery VL, Puri N, Tisherman SA, Vespa PM, Pronovost PJ, Rainey TG, Patterson AJ, Wheeler DS, Task Force on Models for Critical Care (2015). Critical care delivery: the importance of process of care and ICU structure to improved outcomes: an update from the American College of Critical Care Medicine Task Force on Models of Critical Care. Crit Care Med.

