# Supplementary material for: Scientific output and organizational characteristics in Brazilian intensive care units: a multicenter cross-sectional study
Source: Crit Care Sci. 2024 Nov 14;36:e20240006en. doi: 10.62675/2965-2774.20240006-en (PMC11634239; doi:10.62675/2965-2774.20240006-en)
Supplement: Translation

# Translation: Produção científica e características organizacionais em unidades de terapia intensiva brasileiras: um estudo transversal multicêntrico

**DOI:** 10.62675/2965-2774.20240006-pt

**Authors:** Thiago Tavares dos Santos, Luciano César Pontes de Azevedo, Antonio Paulo Nassar, Jorge Ibrain Figueira Salluh

**Affiliations:** 1 São Paulo SP Brasil Instituto de Ensino e Pesquisa, Hospital Sírio-Libanês - São Paulo (SP), Brasil.; 2 São Paulo SP Brasil Hospital Israelita Albert Einstein - São Paulo (SP), Brasil.; 3 São Paulo SP Brasil A. C. Camargo Cancer Center - São Paulo (SP), Brasil.; 4 Rio de Janeiro RJ Brasil Instituto D’Or de Pesquisa e Ensino - Rio de Janeiro (RJ), Brasil.

**Keywords:** Cuidados críticos, Unidades de terapia intensiva, Pesquisa, Qualidade da assistência à saúde, Benchmarking, Brasil

## Abstract

**Objetivo:**

Examinar as associações entre a produção científica das unidades de terapia intensiva brasileiras e suas características organizacionais.

**Métodos:**

Trata-se de uma reanálise de uma coorte retrospectiva anterior, que avaliou as características organizacionais das unidades de terapia intensiva e suas associações com os desfechos. Analisamos dados de 93 unidades de terapia intensiva em todo o Brasil. As unidades de terapia intensiva foram avaliadas quanto à produtividade científica e aos efeitos de suas atividades de pesquisa, usando indicadores de assistência para comparação. Definimos as unidades de terapia intensiva mais produtivas cientificamente como aquelas com várias publicações e escore *SCImago Journal Rank* ou índice h acima dos valores medianos das unidades de terapia intensiva participantes.

**Resultados:**

As unidades de terapia intensiva com mais publicações, pontuações *SCImago Journal Rank* mais altas e escores índice h mais altos tinham número maior de intensivistas certificados (mediana de 7; IIQ 5 - 10 *versus* 4; IIQ 2 - 8; com p < 0,01, na comparação entre unidades de terapia intensiva com mais e menos publicações). As unidades de terapia intensiva com pontuações *SCImago Journal Rank* e índice h mais altas também tiveram um número maior de protocolos totalmente implementados (mediana de 8; IIQ 6 - 8 *versus* 5; IIQ 3,75 - 7,25; p < 0,01, na comparação entre unidades de terapia intensiva com pontuações *SCImago Journal Rank* mais altas *versus* mais baixas).

**Conclusões:**

O trabalho científico foi associado a melhores padrões de pessoal e a maior implementação de protocolos, sugerindo que a atividade de pesquisa pode ser um indicador de melhor organização da unidade de terapia intensiva e da prestação de assistência.

## INTRODUÇÃO

Nas últimas décadas, vários estudos clínicos produziram evidências robustas acerca da prestação de assistência em unidades de terapia intensiva (UTIs).^(1,2)^ No entanto, a tradução da medicina baseada em evidências em desfechos mais favoráveis depende da implementação otimizada dos resultados das pesquisas.^(3-7)^ Além de produzir as evidências necessárias para o desenvolvimento da ciência, há evidências atuais de várias especialidades médicas de que a realização de pesquisas pode melhorar a assistência aos pacientes não diretamente envolvidos nos estudos e, possivelmente, aumentar a qualidade da assistência prestada pelas instituições onde esses estudos são realizados.^(8-12)^

A associação entre a atividade de pesquisa e a qualidade da assistência tem sido demonstrada em especialidades como cirurgia, cardiologia e oncologia.^(13-15)^ Um grande estudo de coorte multicêntrico de pacientes com câncer de ovário demonstrou que o tratamento em centros que não eram de pesquisa estava associado a um maior risco de morte em comparação com o tratamento em instituições de pesquisa.^(13)^ Uma revisão sistemática que avaliou estudos em diversas áreas de pesquisa concluiu que as instituições e os profissionais que participaram de estudos tiveram melhores desfechos clínicos, adesão a diretrizes e maior uso de práticas baseadas em evidências.^(16)^

Nos últimos 30 anos, os desfechos melhoraram significativamente para os pacientes de UTI que seguiram as diretrizes de cuidados baseados em evidências,^(17-20)^ especialmente aqueles que sofrem de insuficiência respiratória aguda, síndrome do desconforto respiratório agudo (SDRA)^(21-23)^ ou sepse.^(24)^ A adesão a protocolos e a estratégias clínicas no tratamento dessas condições está associada a menos complicações adquiridas na UTI e a taxas de mortalidade mais baixas. No entanto, apesar do aumento de pesquisas e de publicações sobre doenças críticas, poucos estudos examinaram as associações entre a atividade de pesquisa clínica e os indicadores de organização da UTI, especialmente em países de baixa e média renda (PBMRs).

Nossa hipótese foi a de que uma maior produção científica está associada a melhores características organizacionais da UTI, ou seja, ao processo de assistência e a alocação de pessoal, em uma grande amostra de UTIs brasileiras. Nosso objetivo foi examinar as associações entre a produção científica das unidades de terapia intensiva brasileiras e suas características organizacionais.

## MÉTODOS

### Desenho do estudo e população

Este estudo é uma análise secundária do estudo *ORganizational CHaractEeriSTics in cRitical cAre* (ORCHESTRA.)^(25)^ O estudo ORCHESTRA consiste em uma coorte multicêntrica de registro contínuo (desde 2013) que visa avaliar as associações entre as características da organização de cuidados críticos e os desfechos de pacientes críticos. O ORCHESTRA II foi realizado em 93 UTIs brasileiras de 55 hospitais durante 2014 e 2015, no qual os dados clínicos dos pacientes e os dados das características da UTI foram coletados com base num inquérito específico.^(25)^ Os Comitês de Ética locais e a Comissão Nacional de Ética em Pesquisa (CAAE: 19687113.8.1001.5249) dispensaram a necessidade de consentimento informado dos pacientes. Foram incluídas no estudo UTIs gerais e especializadas, com exceção daquelas dedicadas exclusivamente a pacientes cardiovasculares.

Todos os dados dos pacientes foram desidentificados e extraídos do *software* Epimed Monitor ICU Database^®^, que é um sistema de gestão de UTI em nuvem para melhoria da qualidade, *benchmarking* e avaliação do conjunto de casos.^(26)^ Os dados coletados rotineiramente incluíam dados demográficos, diagnóstico de admissão, comorbidades segundo o Índice de Comorbidade de Charlson, estado de desempenho na semana anterior à admissão hospitalar, *Simplified Acute Physiological Score 3* (SAPS 3), *Sequential Organ Failure Assessment* (SOFA) na admissão, uso de suporte de órgãos e desfechos na UTI/hospitalares.^(26)^

As características organizacionais dessas UTIs foram coletadas para o estudo ORCHESTRA II^(25)^ por meio de um inquérito estruturado aplicado em 2015 em entrevistas (pessoalmente ou por telefone) com diretores médicos e/ou enfermeiros dos centros participantes. Dados detalhados do ORCHESTRA II estão publicados em outras fontes.^(25)^ Os dados pesquisados incluíram os padrões de pessoal da UTI e um conjunto de dez protocolos clínicos predefinidos destinados a prevenir complicações adquiridas na UTI e a tratar doenças agudas.^(25)^ Também avaliamos a autonomia dos membros da equipe não médica, entrevistando o enfermeiro-chefe, conforme descrito.^(26)^

### Produção científica

Usamos o número de publicações revisadas por pares como um indicador de produção científica. Os dados referentes à produção científica foram obtidos retrospectivamente (em fevereiro de 2020) por meio da elaboração de uma estratégia de busca nos bancos de dados PubMed e Literatura Latino-americana e do Caribe em Ciências da Saúde (LILACS) (Figura 1). O objetivo foi identificar publicações relativas a estudos realizados durante os anos de 2014 e 2015, quando foi realizado o ORCHESTRA II. Assim, considerando um intervalo arbitrário entre a realização da pesquisa e sua publicação, pesquisamos a literatura no período entre 2014 e 2017. Identificamos e registramos o número total de artigos publicados em periódicos científicos revisados por pares. As buscas foram realizadas por um autor nos bancos de dados PubMed e LILACS e foram verificadas por outros autores quando havia alguma incerteza. Considerando a relevância incomparável e a amplitude da cobertura oferecida pelo PubMed e a representatividade local da LILACS, com nossa exigência rigorosa de fontes revisadas por pares, determinamos que estender nossa pesquisa para incluir outros bancos de dados ou literatura cinzenta não aumentaria significativamente a qualidade ou a relevância de nossa pesquisa.

**Figura 1:** Processo de seleção dos artigos.

Os dados referentes à atividade científica foram obtidos retrospectivamente por meio da elaboração de uma estratégia de busca em bancos de dados de publicações revisadas por pares.

Além disso, utilizamos o recurso de pesquisa avançada do PubMed para pesquisar especificamente o nome do hospital associado à UTI do estudo. Isso fez parte de nossa estratégia de pesquisa exploratória mais ampla, com o objetivo de entender completamente o tópico sem nos limitarmos a um protocolo rígido. Essa abordagem flexível permitiu descobrir uma ampla gama de fontes e tipos de informações que um método mais estruturado e sistemático poderia não ter capturado. Ao concentrar nossa busca no hospital de interesse, conseguimos identificar a maior coleção possível de artigos relevantes para nossa pesquisa (Apêndice 1).

Os resultados da pesquisa bibliográfica foram examinados manualmente pelos autores para identificar as publicações e confirmar que suas fontes eram as UTIs participantes do ORCHESTRA.^(25)^ A confirmação incluiu o contato com os autores. No caso de instituições em que havia mais de uma UTI participante do ORCHESTRA II,^(25)^ o nome do coordenador da UTI listado como coautor foi usado para determinar a associação com a publicação. O número total de artigos identificados na pesquisa do PubMed de cada UTI foi adicionado, criando uma variável contínua. O mesmo processo foi realizado no banco de dados LILACS. A qualidade dessas publicações foi avaliada pelo fator de impacto dos periódicos (índice h e *SCImago Journal Rank*)^(27,28)^ no ano da publicação.

### Fatores de impacto e qualificação da produção científica

Avaliamos o impacto das publicações por meio do índice h e do *SCImago Journal Rank* do periódico em que cada artigo foi publicado. O índice h é uma medida da influência científica dos periódicos acadêmicos que considera o número de citações desse periódico e a importância dessas citações. Para a construção da variável contínua do índice h, foi usado o índice h mais alto entre todas as publicações.^(27)^

O escore *SCImago Journal Rank* é uma medida da influência científica de periódicos acadêmicos que considera o número de citações desse periódico e a importância dessas citações. O *SCImago Journal Rank* é uma métrica gratuita de periódicos, uma alternativa ao fator de impacto, e pode ser usada para comparações de periódicos. A soma do *SCImago Journal Rank* de todas as publicações foi calculada para cada UTI, gerando assim variável contínua.^(28)^

Todas as UTIs foram consideradas como tendo pelo menos uma publicação, que foi o artigo índice do estudo ORCHESTRA II.^(25)^

Definimos as UTIs mais produtivas cientificamente como aquelas com várias publicações, escore *SCImago Journal Rank* ou índice h acima dos valores medianos das UTIs participantes.

### Organização e desfechos da unidade de terapia intensiva

Avaliamos indicadores de qualidade da assistência por meio de métricas de estrutura e processo. As medidas foram definidas anteriormente^(25)^ e incluíram o seguinte:

- Medidas de processo: o número de protocolos totalmente implementados.
- Equipe/estrutura: número de médicos certificados pela Associação de Medicina Intensiva Brasileira (AMIB), presença de um farmacêutico clínico e escore de autonomia da enfermagem.

### Análise estatística

Foram aplicadas estatísticas descritivas-padrão e métodos estatísticos inferenciais. As variáveis qualitativas (a presença de um farmacêutico clínico) são apresentadas como distribuições de frequências absolutas e relativas e foram comparadas com o teste do qui-quadrado. As variáveis quantitativas (o número de protocolos totalmente implementados, o número de médicos certificados e o escore de autonomia de enfermagem) são apresentadas como medianas e intervalos interquartis (IIQ) e foram comparadas com o teste de Mann-Whitney.

O erro alfa foi previamente fixado em 5% para a hipótese nula, e o processamento estatístico foi realizado com os programas BioStat versão 5.3 e *Statistical Package for the Social Sciences* (SPSS) versão 27.

## RESULTADOS

As características das UTIs incluídas no estudo ORCHESTRA II estão resumidas na tabela 1. Após a pesquisa inicial no PubMed e a triagem de citações únicas, 415 artigos atenderam aos critérios de inclusão. Considerando que a publicação do artigo do ORCHESTRA II foi pontuada para todas as UTIs participantes, o número médio de publicações por UTI foi de 5,45 artigos (desvio-padrão de 12,79), enquanto a mediana (IIQ) foi 2; IIQ 2 - 4 artigos, respectivamente. O número mínimo de publicações durante o período do estudo foi um, e o máximo foi 101. Em 56 UTIs, houve duas ou menos publicações, e apenas 37 UTIs tiveram mais de duas publicações.

**Tabela 1:** Características dos hospitais participantes no estudo OSCHESTRA II

Tipo de hospital		
	Privado	31 (56)
	Público	10 (18)
	Filantrópico	14 (26)
Leitos hospitalares		234 ± 134.202 (60 - 1.020)
	< 150	21 (38)
	150 - 300	24 (44)
	> 300	10 (18)
Número de UTIs em cada hospital		
	1	20 (36)
	2 ou 3	21 (38)
	> 3	14 (26)
Leitos de UTI/leitos hospitalares*		15,15 ± 6,7
Unidade de cuidados semi-intensivos		
	Sim	21 (38)
	Não	34 (62)
Serviço de emergência		
	Sim	48 (87)
	Não	3 (5)
	Unidade de transferência	4 (7)
Certificação de qualidade da assistência médica†		
	Nenhuma	19 (34)
	Nacional	20 (36)
	Internacional	16 (30)
Times de Resposta Rápida		
	Sim	32 (58)
	Não	23 (42)
Bolsas de estudo de cuidados críticos		
	Sim	36 (66)
	Não	19 (34)

UTI - unidade de terapia intensiva.

* Leitos de unidade de terapia intensiva/leitos hospitalares * 100;

† Organização Nacional de Acreditação (ONA), *Joint Commission International* (JCI) e *Accreditation Canada Internation* (ACI). As variáveis contínuas são expressas como medianas (intervalos) ou média ± desvio-padrão.

As pesquisas realizadas no banco de dados LILACS resultaram em 646 artigos; no entanto, apenas um artigo atendeu aos critérios de inclusão deste estudo. Esse artigo já constava da pesquisa realizada na base de dados PubMed.

Com relação ao impacto, a variável índice h apresentou valor médio de 311,9, mediana de 197 e IIQ 197 - 987. A variável *SCImago Journal Rank* apresentou valor médio de 14.379, com mediana de 7,233 e IIQ 3,94 - 157,4, respectivamente.

As tabelas 2 a 4 descrevem as variáveis de organização das UTIs participantes do estudo e suas associações com a produção científica.

**Tabela 2:** Características da unidade de terapia intensiva segundo o número total de artigos publicados

	Publicações revisadas por pares ≤ 2 (n = 56)	Publicações revisadas por pares > 2 (n = 37)	Valor de p
Número de protocolos de assistência totalmente implementados	5 (4 - 8)	7 (5 - 8)	0,18
Escore de autonomia da enfermagem	6 (4 - 9)	7 (3 - 9)	0,73
Médicos certificados pela Associação de Medicina Intensiva Brasileira	4 (2 - 8)	7 (5 - 10)	< 0,01
Presença de farmacêutico clínico	32 (57,1)	21 (56,7)	0,97

A variável publicação revisada por pares é dicotomizada pela mediana (Grupo 1 = valor menor ou igual à mediana; Grupo 2 = valor maior que a mediana). As variáveis numéricas são apresentadas como medianas (intervalos interquartis), e a presença de um farmacêutico é apresentada como número (%).

**Tabela 3:** Características da unidade de terapia intensiva segundo o escore *SCImago Journal Rank*

	SJR ≤ 7.233 (n = 52)	SJR > 7.233 (n = 41)	Valor de p
Número de protocolos de assistência totalmente implementados	5 (3,75 - 7,25)	8 (6 - 8)	< 0,01
Escore de autonomia da enfermagem	7 (3,75 - 9)	6 (4 - 8)	0,67
Médicos certificados pela Associação de Medicina Intensiva Brasileira	4 (2 - 7)	8 (5 - 13)	< 0,01
Presença de farmacêutico clínico	28 (53,8)	25 (61,0)	0,63

SJR - *SCImago Journal Rank*. A variável *SCImago Journal Rank* é dicotomizada pela mediana (Grupo 1 = valor menor ou igual à mediana; Grupo 2 = valor maior que a mediana). As variáveis numéricas são apresentadas como medianas (intervalos interquartis), e a presença de um farmacêutico é apresentada como número (%).

**Tabela 4:** Características da unidade de terapia intensiva segundo o índice h do periódico em que o artigo foi publicado

	Índice h ≤ 197 (n = 56)	Índice h > 197 (n = 37)	Valor de p
Número de protocolos de assistência totalmente implementados	5 (4 - 7,25)	8 (5 - 8)	0,01
Escore de autonomia da enfermagem	6 (3 - 9)	7 (4 - 9)	0,77
Médicos certificados pela Associação de Medicina Intensiva Brasileira	4 (2 - 7)	8 (5 - 14)	< 0,01
Presença de farmacêutico clínico	30 (53,6)	23 (62,2)	0,54

A variável índice h é dicotomizada pela mediana (Grupo 1 = valor menor ou igual à mediana; Grupo 2 = valor maior que a mediana). As variáveis numéricas são apresentadas como medianas (intervalos interquartis), e a presença de um farmacêutico é apresentada como um número (%).

### Características das unidades de terapia intensiva analisadas considerando volume de publicações, **SCImago Journal Rank** e o índice h do periódico

O grupo de UTIs com publicações acima da mediana do número total de publicações (dois artigos) tinha mais médicos certificados pela AMIB (7; IIQ 5 - 10) do que o grupo de UTIs, com apenas uma publicação (4, IIQ 2 - 8, p < 0,01). Além disso, o grupo de UTIs com publicações em periódicos com escore *SCImago Journal Rank* superior a 7,23 (a mediana do estudo) tinha um número maior de protocolos clínicos implementados (aqueles destinados a prevenir complicações adquiridas na UTI e a tratar doenças agudas), com mediana de oito protocolos (IIQ 6 - 8) *versus* cinco publicações, IIQ 3,75 - 7,25, p < 0,01. Essas UTIs também tinham mais médicos certificados pela AMIB do que o grupo de UTIs com publicações em periódicos com escore *SCImago* inferior à mediana (8, IIQ 5 - 13,4, IIQ 2 - 7, p < 0,01).

O grupo de UTIs que teve publicações em periódicos com índice h maior do que a mediana de 197 teve um número maior de protocolos de cuidados implementados (8, IIQ 5 - 8 *versus* - 5, IIQ25 - 75% 4 - 7,25, p < 0,01) e mais médicos certificados pela AMIB (8, IIQ 5 - 14 *versus* 4, IIQ 2 - 7, p < 0,01).

## DISCUSSÃO

Neste amplo estudo multicêntrico de UTIs brasileiras, demonstramos que as UTIs com maior produção científica tinham mais protocolos clínicos implementados e melhores padrões de pessoal (ou seja, mais intensivistas) do que as UTIs com menor produção científica.

Neste estudo, usamos substitutos de processos e organizações alinhados àqueles que são atualmente recomendados^(29-31)^ e que foram amplamente validados.^(25,26,32-35)^ Soares et al. demonstraram, em estudo com 78 UTIs brasileiras, que a implementação de protocolos por uma equipe multidisciplinar (uma combinação de processo e coordenação de cuidados pela equipe da UTI) estava associada a melhores taxas de sobrevida.^(17)^ Além disso, há evidências de que uma melhor equipe de UTI e a implementação de protocolos estão fortemente associadas a uma melhor adesão às metas de ventilação protetora^(36)^ e sedação em pacientes em ventilação mecânica.^(37)^

Curiosamente, nossos resultados demonstram que, nas UTIs envolvidas em pesquisas clínicas, os possíveis benefícios organizacionais e de desfecho são sistêmicos e não se limitam aos pacientes que participam de estudos clínicos. Obviamente, é legítimo argumentar que as UTIs com melhor equipe são possivelmente aquelas com mais recursos e, portanto, estarão mais propensas a realizar pesquisas clínicas. Acreditamos que melhores processos de cuidado (implementação de protocolos) demonstram que, além das relações causais, as UTIs envolvidas em pesquisas estão envolvidas em um processo de otimização dos padrões de cuidado (equipe e protocolos baseados em evidências). Essas condições estão, em última análise, associadas a melhores desfechos.

Embora não tenham sido descritos anteriormente em UTIs, nossos resultados estão condizentes com as evidências encontradas em outras especialidades, como cardiologia, neurologia e oncologia. Nesses cenários, a conformidade com os indicadores de qualidade da assistência foi superior em ambientes de assistência com maior atividade científica. Como exemplos, podemos citar o manejo da síndrome coronariana aguda, o tratamento da neoplasia ovariana e as melhorias no diagnóstico de síndromes neurológicas clínicas.^(13-15)^

Nosso estudo tem vários méritos. Trata-se de um estudo multicêntrico de grande porte envolvendo UTIs em um país de renda média-alta, no qual capturamos padrões organizacionais que foram previamente validados como associados à qualidade da assistência e aos desfechos em UTIs em geral^(25)^ e em ambientes específicos.^(37,38)^

No entanto, o presente estudo tem também várias limitações. Em primeiro lugar, há uma falta de consenso na literatura sobre métodos para medir a produtividade e a produção científica, a natureza retrospectiva do modelo metodológico utilizado e o viés de seleção inerente, pois as UTIs participantes eram aquelas que já colaboravam em algum nível em estudos multicêntricos. Dessa forma, não tivemos UTIs sem nenhuma inserção acadêmica ou produção científica como parâmetro de comparação. Entretanto, podemos levantar a hipótese de que, se tais diferenças estiverem presentes entre os envolvidos em pesquisas, elas seriam ainda maiores se outros centros fossem incluídos. Além disso, não medimos o impacto das atividades de pesquisa clínica, como a taxa de mortalidade padronizada e os desfechos dos pacientes, nas medidas de desempenho da UTI; portanto, não foi possível demonstrar uma associação direta entre pesquisa clínica e prognóstico mais favorável.

## CONCLUSÃO

As unidades de terapia intensiva brasileiras envolvidas em pesquisa clínica, demonstradas pelo aumento do número de publicações e publicações com pontuações mais altas nos escores *SCImago Journal Rank* e índice h, têm melhores padrões de pessoal e implementação de protocolos clínicos. Esses achados confirmam a importância de investir na organização da unidade de terapia intensiva e, ao mesmo tempo, criar condições adequadas para o avanço do conhecimento por meio da pesquisa clínica.

## Apêndice 1 Estrutura de busca em banco de dados

**Table:**

Hospital	Estrutura de busca
Hospital Agenor Paiva - Salvador (BA), Brasil	"Hospital Agenor Paiva" OR "Agenor Paiva"
Hospital Santa Helena - Camaçari (BA), Brasil	"Hospital Santa Helena" OR ("Hospital Santa Helena") AND ("Camaçari") OR "Hospital Santa Helena, Camaçari"
Hospital Anchieta Distrito Federal - Taguatinga (DF), Brasil	"Hospital Anchieta Distrito Federal, Taguatinga" OR "Hospital Anchieta" OR "Hospital Anchieta Distrito Federal"
Hospital Brasília - Brasília (DF), Brasil	"Hospital Brasília"
Hospital Santa Luzia Rede D’Or São Luiz DF - Brasília (DF), Brasil	"Hospital Santa Luzia Rede D’Or São Luiz DF" OR "Hospital Santa Luzia Rede D’Or" OR "Hospital Santa Luzia" OR "Santa Luzia Rede D’Or"
Hospital Unimed Vitória - Vitória (ES), Brasil	"Hospital Unimed Vitoria" OR "Unimed Vitoria"
Hospital Geral de Goiânia - Goiânia (GO), Brasil	"Hospital Geral de Goiânia" OR "Hospital Geral Goiânia"
Hospital de Câncer do Maranhão Tarquínio Lopes Filho - São Luís (MA), Brasil	"Hospital de Câncer do Maranhão Tarquínio Lopes Filho" OR "Hospital Maranhão Tarquínio Lopes Filho"
UDI Hospital - São Luís(MA), Brasil	"Hospital UDI" OR "UDI hospital"
Santa Casa de Caridade de Diamantina - Diamantina (MG), Brasil	"Santa Casa Diamantina" OR "Caridade de Diamantina"
Hospital das Clínicas, Universidade Federal de Minas Gerais - Belo Horizonte (MG), Brasil	"Hospital" OR "Hospital das Clínicas" AND "universidade Federal de Minas Gerais"
Hospital Universitário Lauro Wanderley - João Pessoa (PB), Brasil	("Hospital Universitário Lauro Wanderley" OR "Hospital Lauro Wanderley" OR "Lauro Wanderley" OR ("Hospital Universitário da Universidade Federal da Paraiba")
Hospital Esperança - Recife (PE), Brasil	"Hospital Esperança"
Hospital Esperança Olinda - Olinda (PE), Brasil	"Hospital Esperança Olinda"
Hospital São Marcos - Recife (PE), Brasil	"Hospital São Marcos"
Hospital Estadual Getúlio Vargas - Rio de Janeiro (RJ), Brasil	"Hospital Estadual Getúlio Vargas" OR "Hospital Getúlio Vargas"
Clínica São Vicente - Rio de Janeiro (RJ), Brasil	"Clínica São Vicente"
Hospital Estadual Adão Pereira Nunes - Duque de Caxias (RJ), Brasil	"Hospital Estadual Adão Pereira Nunes"
Hospital Estadual Carlos Chagas - Rio de Janeiro (RJ), Brasil	"Hospital Estadual Carlos Chagas"
Hospital Quinta D’Or - Rio de Janeiro (RJ), Brasil	((("Hospital Quinta D’Or") OR ("Hospital Quinta Dor")) OR ("Quinta D’Or")) OR ("Quinta Dor")
Hospital Barra D’Or - Rio de Janeiro (RJ), Brasil	((("Hospital Barra D’Or") OR ("Hospital Barra Dor")) OR ("Barra D’Or")) OR ("Barra Dor")
Hospital Copa D’Or - Rio de Janeiro (RJ), Brasil	((("Hospital Copa D’Or") OR ("Hospital Copa Dor")) OR ("Copa D’Or")) OR ("Copa Dor")
Hospital Caxias D’Or - Duque de Caxias (RJ), Brasil	((("Hospital Caxias D’Or") OR ("Hospital Caxias Dor")) OR ("Caxias D’Or")) OR ("Caxias Dor")
Hospital Norte D’Or - Rio de Janeiro (RJ), Brasil	((("Hospital Norte D’Or") OR ("Hospital Norte Dor")) OR ("Norte Dor")) OR ("Norte D’Or")
Hospital Oeste D’Or - Rio de Janeiro (RJ), Brasil	"Hospital Oeste D’Or" OR "Hospital Oeste Dor" OR "Oeste D’Or" OR "Oeste Dor"
Hospital Rios D'Or - Rio de Janeiro (RJ), Brasil	((("Hospital Rios D’Or") OR ("Hospital Rios Dor")) OR ("Rios D’Or")) OR ("Rios Dor")
Hospital Municipal Souza Aguiar, Rio de Janeiro (RJ), Brasil	"Hospital Municipal Souza Aguiar" OR "Souza Aguiar" OR "Hospital Souza Aguiar" OR "HMSA"
Hospital São Lucas - Rio de Janeiro (RJ), Brasil	"Hospital São Lucas" OR "São Lucas"
Hospital Unimed Costa do Sol - Campos dos Goytacazes (RJ), Brasil	"Hospital Unimed Costa do Sol" OR "Hospital Costa do Sol" OR "Hospital Costa do Sol"
Hospital Niterói D’Or - Niterói (RJ), Brasil	"Hospital Niteroi D’Or" OR "Hospital Niteroi Dor" OR "Niteroi D’Or" OR "Niteroi Dor"
Hospital Israelita Albert Sabin - Rio de Janeiro (RJ), Brasil	"Hospital Israelita Albert Sabin" OR "Israelita Albert Sabin" OR "Hospital Albert Sabin"
Samer Hospital - Resende (RJ), Brasil	"Samer Hospital, Resende" OR "Samer"
Hospital Estadual Alberto Torres - São Gonçalo (RJ), Brasil	"Hospital Estadual Alberto Torres" OR "Hospital Alberto Torres" OR "Alberto Torres"
Instituto Nacional de Câncer - HC II - Rio de Janeiro (RJ), Brasil	"Instituto Nacional de Câncer" OR "INCA"
Hospital Badim - Rio de Janeiro (RJ), Brasil	"Hospital Badim" OR "Badim"
Hospital Santa Rita, Santa Casa de Misericórdia de Porto Alegre - Porto Alegre (RS), Brasil	("Santa Casa de Misericórdia de Porto Alegre") OR ("Hospital Santa Rita")
Pavilhão Pereira Filho, Santa Casa de Misericórdia de Porto Alegre - Porto Alegre (RS), Brasil	(("Santa Casa de Misericórdia de Porto Alegre") AND ("Pavilhão Pereira Filho")) OR (("Pavilhão Pereira Filho"))
Hospital Dom Vicente Scherer, Santa Casa de Misericórdia de Porto Alegre - Porto Alegre (RS), Brasil	(("Santa Casa de Misericórdia de Porto Alegre") AND ("Hospital Dom Vicente Scherer")) OR (("Hospital Dom Vicente Scherer"))
Hospital Montenegro - Montenegro (RS), Brasil	"Hospital Montenegro"
Hospital São Francisco, Ribeirão Preto (SP), Brasil	"Hospital São Francisco" AND "Ribeirão Preto"
Hospital Vivalle - São José dos Campos (SP), Brasil	"Hospital Vivalle"
Fundação Pio XII - Hospital de Câncer de Barretos – Barretos (SP), Brasil	"Hospital de Câncer de Barretos" OR "Fundação Pio XII"
Hospital Alemão Oswaldo Cruz - São Paulo (SP), Brasil	"Hospital Alemão Oswaldo Cruz" OR "Hospital Oswaldo Cruz"
Hospital Israelita Albert Einstein - São Paulo (SP), Brasil	("Hospital Israelita Albert Einstein") OR ("Hospital Albert Einstein")
Hospital Sírio Libanês - São Paulo (SP), Brasil	"Hospital Sírio Libanês" OR "Sírio Libanês"
Hospital São Luiz - Unidade Assunção - São Bernardo do Campo (SP), Brasil	"Hospital São Luiz" AND "Unidade Assunção"
Hospital Sepaco - São Paulo (SP), Brasil	"Hospital Sepaco"
Hospital Santa Paula - São Paulo (SP), Brasil	"Hospital Santa Paula"
Hospital do Rim - São Paulo (SP), Brasil	"Hospital do Rim" OR "Hrim"
Rede D’Or São Luiz - Unidade Itaim - São Paulo (SP), Brasil	(((("Rede Dor São Luiz" OR "Itaim")) OR ("Rede D’Or São Luiz" OR "Itaim")) OR ("São Luiz" OR "Itaim")) OR ("Rede D’Or" OR "Itaim")) OR ("Rede Dor" OR "Itaim")
Hospital Samaritano - São Paulo (SP), Brasil	"Hospital Samaritano"
Hospital do Coração – HCor - São Paulo (SP), Brasil	"Hospital do Coração" OR "HCor"
Hospital Nove de Julho - São Paulo (SP), Brasil	"Hospital Nove de Julho" OR "Hospital 9 de Julho"
Hospital da Luz - Vila Mariana - São Paulo (SP), Brasil	"Hospital da Luz"
Rede D’Or São Luiz - Unidade Morumbi - São Paulo (SP), Brasil	(((("Rede Dor São Luiz" AND "Morumbi")) OR ("Rede D’Or São Luiz" AND "Morumbi")) OR ("São Luiz" AND "Morumbi")) OR ("Rede D’Or"AND "Morumbi")) OR ("Rede Dor" AND "Morumbi")

Em todas as buscas foram aplicados filtros de tempo (período de 2014 a 2017) e idioma (inglês).
